# The Role of Cold Atmospheric Plasma in Wound Healing Processes in Critically Ill Patients

**DOI:** 10.3390/jpm13050736

**Published:** 2023-04-26

**Authors:** Tatiana Bolgeo, Antonio Maconi, Menada Gardalini, Denise Gatti, Roberta Di Matteo, Marco Lapidari, Yaroslava Longhitano, Gabriele Savioli, Andrea Piccioni, Christian Zanza

**Affiliations:** 1 Department of Integrated Research and Innovation Activities, AON SS. Antonio e Biagio e Cesare Arrigo, 15121 Alessandria, Italy; 2 Department of Vascular Surgery, St. Antonio and Biagio and Cesare Arrigo Hospital, 15121 Alessandria, Italy; 3 Department of Anesthesiology and Perioperative Medicine, University of Pittsburgh, Pittsburgh, PA 15260, USA; 4 Department of Emergency Medicine and Surgery, IRCCS Fondazione Policlinico San Matteo, 27100 Pavia, Italy; 5 Department of Emergency Medicine, Fondazione Policlinico Universitario A. Gemelli IRCCS, Università Cattolica del Sacro Cuore, 00168 Rome, Italy

**Keywords:** cold atmospheric plasma, wound, critical care, infection

## Abstract

Critically ill patients are at risk of skin wounds, which reduce their quality of life, complicate their pharmacological regimens, and prolong their hospital stays in intensive care units (ICUs), while also increasing overall mortality and morbidity rates. Cold atmospheric plasma (CAP) has been proposed as a viable option for many biological and medical applications, given its capacity to reduce wound bacterial contamination and promote wound healing. The aim of this narrative review is to describe how CAP works and its operating mechanisms, as well as reporting its possible applications in critical care settings. The success of CAP in the treatment of wounds, in particular, bedsores or pressure sores, presents an innovative path in the prevention of nosocomial infections and an opportunity of reducing the negative implications of these diseases for the NHS. This narrative review of the literature was conducted following the ‘Scale for the Assessment of Narrative Review Articles’ (SANRA) methodology. Previous literature highlights three biological effects of plasma: inactivation of a wide range of microorganisms, including those that are multi-drug-resistant; increased cell proliferation and angiogenesis with a shorter period of plasma treatment; and apoptosis stimulation with a longer and more intensive treatment. CAP is effective in many areas of the medical field, with no significant adverse effects on healthy cells. However, its use can produce potentially serious side effects and should, therefore, be used under expert supervision and in appropriate doses.

## 1. Background

Intensive care units (ICUs) are specialized hospital facilities where critically ill patients with high mortality and morbidity risks are treated.

ICU patients may have skin injuries due to trauma or accidents, such as burn wounds, or due to other causes. They can also be the outcome of primary diseases or their complications, as well as clinical circumstances, along with complex therapy regimens [1].

Trauma patients admitted to intensive care units may present different types of skin injuries, ranging from abrasions and excoriations to penetrating wounds, puncture wounds, cutting wounds, blunt-force trauma wounds, lacerated wounds, and lacerated-contused wounds [2], all possibly due to different causes, such as traffic accidents, stab wounds, falls, etc.

Trauma accounts for 4.3 million annual deaths globally, with road traffic injuries prevailing among adolescents and young adults, while falls predominate among the elderly [3].

Burn injuries are the most common and destructive type of trauma caused by thermal, chemical, electrical, or radiation contact; they are classified based on their depth (first-degree, superficial burns; second-degree, partial-thickness burns; and third-degree, full-thickness burns) and the percentage of total body area affected [4].

According to the WHO, 11 million burns of all types occur worldwide each year, of which 180,000 fatal [5]. Patients with severe burns (>20% of the total body surface area) will require ICU hospitalization to reduce the risk of mortality [6].

Another significant challenge for these types of trauma patients in ICUs is health care-associated infections (HCAIs), which are infections acquired while seeking treatment for medical or surgical conditions and are the most common adverse event during care delivery [7].

HCAIs are a severe issue for patient safety, and their consequences might include longer hospitalizations, long-term disabilities, increased antimicrobial agent resistance, a significant additional financial burden for the health system, high expenditures for individuals and their families, and a mortality increase [8,9]. In Europe, HCAIs cause 16 million extra hospital days and almost 37,000 fatalities, with an annual cost of 7 billion euros [10].

With an incidence ranging from 3.3% to 52.9% [11], critically ill patients, particularly those with HCAIs, are at high risk of developing pressure injuries; the causes are related to prolonged hospitalization, immobility, deep sedation, the use of vasoactive drugs, hypotension, anasarca, organ dysfunction, and altered nutritional status [12].

Pressure sores are areas of localized skin damage and underlying tissues, caused by pressure, friction, and cutting. Their predisposing factors are related to the patient’s characteristics (hypo-trophy, malnutrition, diabetes, hypo-perfusion, skin constantly exposed to moisture, altered sensory perception, limited mobility, and age) [13], as well as factors directly related to the care procedures adopted during hospitalization (prolonged bed rest, drug side-effects, and surgical operations) [14].

Pressure sores are a clinically significant issue. Their occurrence can impair functional recovery and lead to infectious challenges, as they create a breeding ground for bacterial super infections that can be localized or escalate to generalized sepsis, lengthening hospitalizations [15] as well as increasing morbidity and mortality [16].

These patients also require the use of modern monitoring and treatment techniques, both invasive and noninvasive, which can result in varying degrees of skin damage. Complications caused by medical devices are related to their capacity to induce infections [17] and also issues related to their implementation [18]. Wounds caused by infectious agents are the most common in intensive care settings and occur during severe diseases. Bed rest, moisture, and obesity all contribute to inflammatory dermatitis in the major skin folds, with stratum corneum damage promoting the entrance of germs such as Candida albicans or beta-hemolytic Streptococcus bacteria [19].

Management of skin wounds in critically ill patients is crucial, as these wounds reduce quality of life, complicate treatment regimens, lengthen ICU stays, and increase mortality and morbidity [20,21].

Cold atmospheric plasma (CAP) has been proposed as an option for many different biological and medical applications as a result of its ability to reduce the bacterial count within a wound and promote wound healing.

Plasma is a form of ionized gas that has a high concentration of charged particles (OH^−^, H_2_O^+^, and electrons), reactive chemicals (oxygen free radicals, or “ROS,” and reactive nitrogen species, or “RNS”), excited molecules, and UV photons (UVB, UVC) [22]. The physical-chemical properties of plasma are affected by a variety of factors, including the kind of gas or mixture of gases employed and the applied energy, pressure, and atmosphere [23].

The use of physical plasma enables two approaches: the use of plasma-based or plasma-integrated procedures to treat surfaces/materials/devices in order to apply particular dressings and the direct application of physical plasma in the human or animal body in order to use its therapeutic benefits [23]. Its application is reported in a large and growing number of articles that consider it a very promising therapy [24,25].

The aim of this narrative review is to discuss the functioning mechanisms of CAP treatment and to report on its potential uses in the critical care setting. As a result, this review focuses on research that has addressed situations common in critically ill patients, such as acute wounds, burn wounds, skin grafts, and secondary injuries such as pressure sores.

## 2. Methods

A narrative review of the literature was conducted [26] following the ‘Scale for the Assessment of Narrative Review Articles’ (SANRA) methodology. It involves 6 stages: (1) justify the importance of the review; (2) state the specific purposes or pose research questions; (3) describe the adopted research strategy; (4) attribute references to the summarized statements; (5) demonstrate scientific reasoning in the discussion; and (6) report data appropriately [27].

At the initial stage, the following research questions were raised:

- What are the working mechanisms of CAP therapy on wounds in critically ill patients?
- What are the implications of CAP to date, related to wounds in critically ill patients, as reported in the literature?

The literature research was conducted between June and September 2022 using PubMed, Scopus, and Cochrane Central Register databases. The keywords CAP, wound, critical care, and infection were searched as MeSH or major terms or as accessible terms and were related in various ways, depending on the syntax of the databases consulted. For review purposes, the following studies were included: (a) in English or Italian, (b) conducted using whichever research methodology, and (c) both primary and secondary sources.

No time limits were imposed in relation to the date of publication. After reviewing the literature, two authors (R.D. and M.G.) made the article selections, and any doubts were resolved by consulting with a third researcher (D.G.). The key data from the included studies were extracted into an Excel^®^ sheet in line with the research questions, analyzed, and debated as a group. Subsequently, the researchers developed a narrative presentation of the results that was divided into three macro-areas concerning (a) the characteristics of CAP; (b) the reduction in wound area; and (c) the reduction in bacterial load.

## 3. Results

### 3.1. Characteristics of Cold Atmospheric Plasma

In physics, plasma is referred to as the fourth state of matter (after the solid, liquid, and gas phases), or ionized gas, depending on its temperature, and it can be divided into hot plasma and cold plasma. Plasma can be distinguished into standard (“thermal”) plasma, at 4000–5000 K, and low-temperature (“cold” or “non-thermal”) plasma, at 30–50 °C, which generates oxygen and nitrogen free radicals with positive and/or negative ions [28].

Since high temperatures can cause thermal damage to organisms, the medical field must consider a type of plasma at atmospheric pressure with its temperature close to room temperature [29]. To be considered “plasma,” a gas must meet certain criteria, including being molecularly neutral (or near-neutral), having a Debye shield (charged particles capable of counteracting an electrostatic field within a Debye), and having a plasma frequency, defined as the natural oscillation frequency that determines particle movement, causing the gas to return to its neutral state [30].

The main components of CAP include ions, electrons, metastables, photons, and electromagnetic fields. After a reaction with environmental air, CAP forms a hierarchical group of reactive oxygen and nitrogen species (RONS) that promote increased skin tissue microcirculation, increased monocyte stimulation, increased cell migration, and stimulation of the keratinocytes and fibroblasts primarily involved in wound healing.

Plasma can be applied directly or indirectly. In the former case, cell lines receive plasma discharges in vitro and animal or human tissue in vivo; in the latter case, a plasma-activated solution is used [30]. To improve CAP efficiency, helium (He), argon (Ar), nitrogen (N_2_), oxygen (O_2_), artificial air, and two or more mixtures of these gases can be used to generate CAP [31].

Energy is required to produce and maintain the plasma. Several devices have been developed in the biomedical field that use electrical energy. Some methods used to produce CAP include: dielectric barrier discharge (DBD), atmospheric-pressure plasma jets (APPJ), plasma needles, and plasma pencils [32].

### 3.2. Wound Area Reduction

Wound healing is a complex process involving four distinct phases: hemostasis, inflammation, skin proliferation, and remodeling [33]. Wounds can typically be classified as acute and chronic wounds. Acute wounds include abrasions, scalds, burns, or postoperative incisions; chronic wounds do not heal in an orderly manner, frequently remaining in the inflammatory phase for too long and sometimes being complicated by systemic diseases, age, and repeated trauma, such as diabetic ulcers, venous ulcers, arterial ulcers, and pressure sores [34].

CAP may promote wound healing through its antiseptic effects, stimulating the proliferation and migration of skin cells by activating or inhibiting integrin receptors on the cell surface or through its pro-angiogenic effect [35]. It also appears to act by triggering the production of nitric oxide (NO), which promotes cell migration and the assembly of endothelial cells into vessel-like structures useful for wound neo-vascularization [36].

Treatment with CAP can be adapted according to the different stages of wound healing; argon plasma was found to be better in promoting coagulation, while helium plasma was more effective in healing [37].

**Figure 1:**
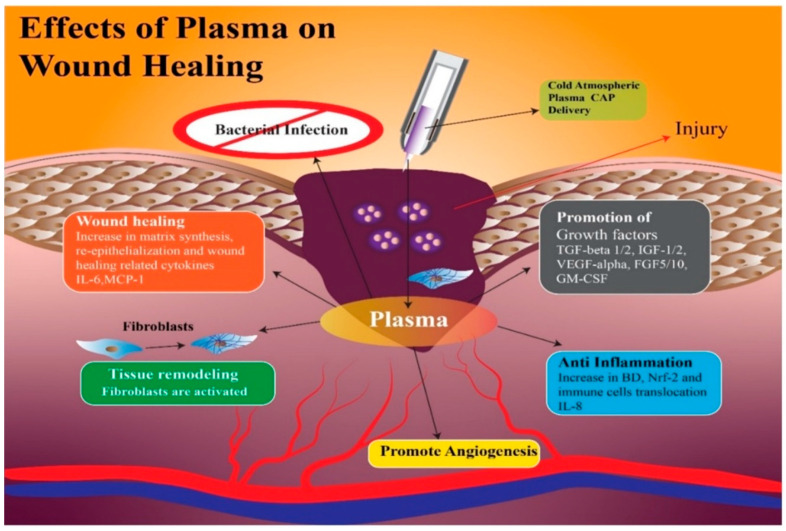
With the application of CAP on the wound, there is an increase in growth factors such as Tumor Growth Factor-β 1 and 2; Insulin Growth Factor 1 and 2; Vascular-Endothelial Growth Factor-α; Granulocyte-Macrophage Colony-Stimulating Factor, which facilitate angiogenesis with the anti-inflammatory effect of interleukin-8. Finally, the wound heals with re-epithelialization and synthesis of a new matrix, thanks to the fibroblasts and cytokines, Membrane Cofactor Protein-1 and Interleukin-6.

**Figure 2:**
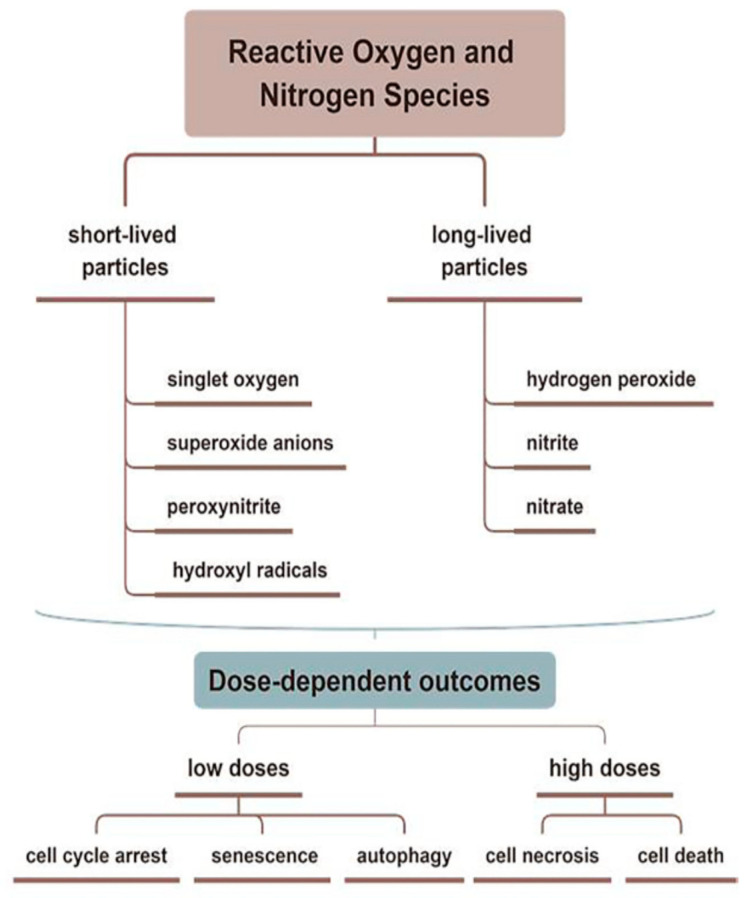
Reactive oxygen and reactive nitrogen species (ROS and RNS) related to CAP are divided into short- and long-lived molecules, which play different roles in the treating skin diseases. CAP promotes wound healing through antiseptic and pro-angiogenic effects, stimulating the proliferation and migration of skin cells by activating/inhibiting integrin receptors.

Amini et al. [38] demonstrated that treatment with CAP modifies the persistence levels of inflammatory cytokines and growth factors including IL-1, IL-8, TGF-β, TNF-α, and INF-γ, promoting healing through a more rapid initiation of the proliferative phase. In addition, CAP generates reactive oxygen species (ROS) and nitrogen species (RNS), which can increase the synthesis of pro-angiogenic factors, consequently promoting wound healing [39] (Figure 1 and Figure 2).

The duration of CAP therapy should be carefully monitored during wound treatment, as overdosage could cause necrosis or apoptosis, invalidating the wound healing process [40].

Gao et al. [41] studied the effect of CAP on two traumatic wounds. The authors employed plasma equipment, treating patients as a counter-electrode, with a power of roughly 50 mW to study the impact of CAP on two traumatic wounds. The first patient had utilized ineffective self-treatment, resulting in secondary eczema with exudation and scab development. Treatment with CAP for 20 min every other day prevented the wound exudation; wound healing was visible after three consecutive treatment cycles. The wound of the second patient had been treated unsuccessfully with antibiotics, and complete wound healing was noted following three consecutive cycles of CAP administration.

This research also included a patient with a big genital wart that was treated with CAP; the ablation site was treated with CAP for 40 min after the wart was removed, and this was repeated three days later; full healing became apparent after two re-applications. In another study [42], CAP direct application was used on 27 wounds (19 hard-to-heal wounds and 8 acute wounds), with an average wound area of 15 cm^2^, for 180 s three times a week in addition to standard therapy. All acute wounds and 68% of the hard-to-heal wounds healed after an average treatment period of 14 weeks; the therapy was only not effective on two hard-to-heal wounds.

Additionally, skin grafting is a unique procedure that removes healthy skin from an area of the body and transplants it into a damaged area. Heinlin et al. [43] enrolled 40 patients to evaluate the impact of cold atmospheric argon plasma on the healing process of the donor site of skin grafts on the upper leg. The investigated wound sites were separated into two distinct areas of the same size and received either CAP indirect therapy or a placebo at random (argon gas) for 2 min. From the second day of treatment, positive effects in terms of improved re-epithelialization, reduction in fibrin layers, and blood crusts were observed in the plasma-treated areas, compared with the placebo-treated areas.

In the recipient site, Frescaline et al. found that CAP could improve extracellular matrix (ECM) formation through activation of the canonical TGF-β1 SMAD-dependent pathway [44] (Figure 1).

An experimental study investigating the effects of argon-jet plasma on donor sites of skin grafts in an environment that was not protein-colonized by bacteria found a positive outcome on wound healing, compared with untreated controls [43].

CAP has also been used to treat burn wounds, hypothesizing an increase in angiogenesis as a determinant of healing. In the study by Betancourt-Angeles et al. [45] on the treatment of human burn wounds, a reduction in pain and itching was found after a three-minute helium CAP application; an extra three-minute treatment after 16 h significantly accelerated healing and the development of new tissue. The authors did not analyze molecular pathways during or after CAP administration since this research was a case report.

The ICU patient very often has wounds due to protracted immobility, critical conditions, or comorbidities, which can cause further complications [46]. Nguyen et al. [47] treated wounds (soft tissue skin lesions, burns, pressure ulcers, shingles, contact, and atopic dermatitis) with CAP at all stages in patients admitted to the ICU with severe COVID-19 infections. A total of 70% of patients who underwent irradiation with CAP as a supportive treatment showed complete epithelialization after 14 days; in addition, cessation of exudation and a reduction in wound pain were noted. The authors point out that in the period before they introduced CAP, these wounds took longer to heal.

Considering the effects of CAP on the activation of biological processes to promote the regeneration and proliferation of various cell types, CAP has also been studied in neuronal regeneration; according to some studies, CAP could promote in vitro differentiation of neuronal stem cells and protect against oxidative stress [48,49].

**Figure 3:**
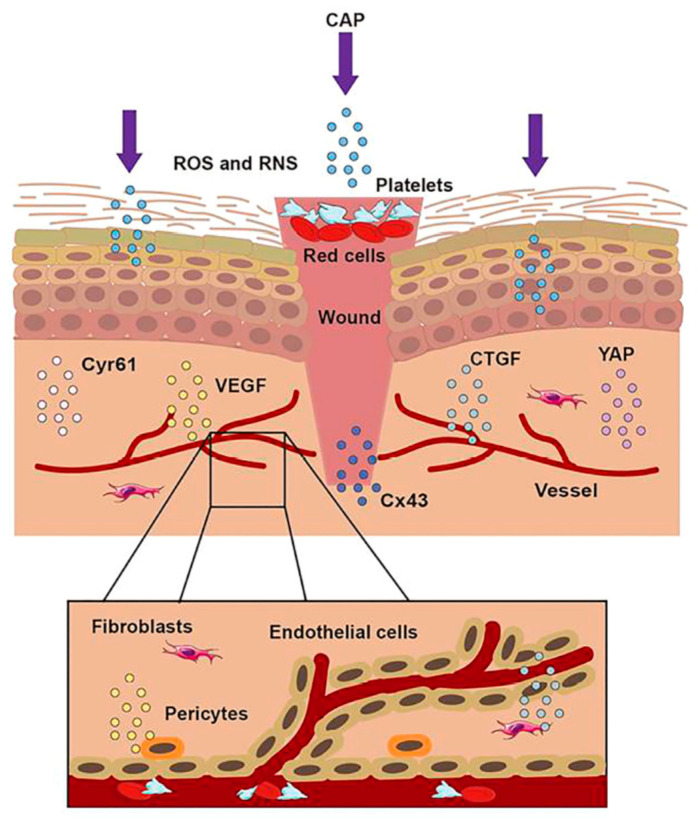
The treatment of CAP on a wound. When the skin was injured, the first step was to form a blood scab to protect the wound. CAP could accomplish wound healing through short-lived and long-lived ROS and RNS. CAP could promote the formation of new blood vessels, strengthen the release of Connective Tissue Growth Factor (CTGF) and Vascular Endothelial Growth Factor (VEGF), activate the Yes-Associated Protein (YAP) pathway, and upregulate the expression of Connexin 43 (Cx43) and Cysteine-rich angiogenic inducer 61 (Cyr61).

Nervous system trauma and neurodegeneration often result in permanent functional deficits due to the limited regenerative capacity of the brain and spinal cord. It is known that the central nervous system (CNS) has a limited ability to regenerate following an injury or neurodegenerative disease [50], with a major impact on quality of life for patients. Kativar et al. [51] concluded that astrocytes, glial cells that support neurons through protein secretion of and neurotrophic factors, can respond to properly calibrated nanosecond-pulse dielectric discharge plasma treatment, which can directly promote their growth and improve their ability to enhance neuronal regeneration after an injury. It is interesting to note the importance of the therapeutic dose on responses: low intensities (≤10 mJ) caused no measurable changes, while high intensities (≥90 mJ) generally resulted in widespread cell death. Intermediate intensities (10–50 mJ) elicited a physiological response, resulting in improved cell regeneration (Figure 1 and Figure 3)

### 3.3. Bacterial Load Reduction

CAP’s bactericidal activity has received 20 years of attention and investigation [50]. The potential uses of CAP for infection control in clinical settings have grown significantly during the last two decades. The ability of CAP to effectively eradicate bacterial biofilms has been demonstrated by several bacterial studies [51,52,53,54]. This is what inspired the concept of utilizing CAP to lower the bacterial wound burden and so improve healing [55]. Early published studies employed jet plasma using argon as the carrier gas. In 2010, a prospective study used argon plasma treatment for 5 min via a CAP device called MicroPlaSter alpha, which resulted in a very significant reduction in bacterial load, compared to standard wound care alone [55]. In fact, after the application of CAP, a mean reduction of 1.10 log_10_ was observed in the intervention group and a reduction of 0.41 log_10_ in the control group. Although the difference in the mean reduction between the two arms of the study is significant, an intervention that achieves a bacterial reduction of 1 log_10_ would hardly be considered relevant as “bactericidal” or “antimicrobial.” In fact, general agreement indicates that the effective bactericidal action of a device refers to a reduction of at least 3 log_10_ in the number of viable bacterial cells tested [56,57]. A following study used a second-generation device known as the MicroPlaSter beta, which has a flexible four-joint therapy arm which enables treatment of difficult-to-reach areas. A patient with Hailey-Hailey illness (also known as benign chronic pemphigus) and secondary infections with Candida albicans and Proteus mirabilis saw rapid clinical recovery. Another study [58] clearly demonstrates that a short CAP treatment of 2 min is sufficient to achieve significant reduction in the bacterial load on chronic infected wounds in vivo. Efficacy and tolerability were demonstrated in both generations of devices (MicroPlaSter alpha and MicroPlaSter beta). These studies treated venous, arterial, diabetic, and traumatic ulcers, and reduced bacterial infection was observed regardless of bacterial type.

Maisch et al. [54] reported the efficacy of CAP treatment in decolonizing methicillin-resistant *Staphylococcus aureus* (MRSA) and *Escherichia coli* without causing any cellular damage.

Subsequent study results suggest that CAP could be effective for different types of bacteria, including gram-positive and gram-negative bacteria, anaerobes, aerobes, or facultative anaerobes [59]. However, some studies have found resistance of some bacteria to CAP [60,61]. The response of bacteria to CAPs is species-dependent, and gram-positive bacteria have shown greater resistance, indicating the importance of cell wall thickness for the CAP-mediated inactivation time [62]. CAP treatment has been shown to be effective in inhibiting beta-hemolytic streptococci and *S. aureus* [63], gram-positive aerobic cocci mainly responsible for acute bacterial skin infections [64].

Infection in burn wounds is the most serious consequence of this type of trauma due to the delay in wound recovery and healing; it can also cause bacteremia, sepsis, and multi-organ dysfunction syndromes in the most severe cases, resulting in increased mortality, disease exacerbation, and increased health care burdens [6].

A case report describes the state of a man who suffered two lower-extremity wounds from second-degree burns produced by boiling oil, one with a damaged area of 15 cm^2^ and the other with a damaged area of 79 cm^2^. The wounds were inflamed, and the patient experienced severe pain. Three hours after the first treatment with CAP, it was repeated on both wounds for three minutes each. The patient, 16 h after the application of the second treatment, reported no discomfort, whereas a process of re-epithelialization of both wounds and an absence of bacterial infection were observed [45].

Furthermore, CAP displays antibacterial activity via electrostatic stress [65,66]; Das et al.’s [67] findings suggest that the oxidative stress induced by reactive oxygen and nitrogen species could play a key role in bacterial inactivation.

**Figure 4:**
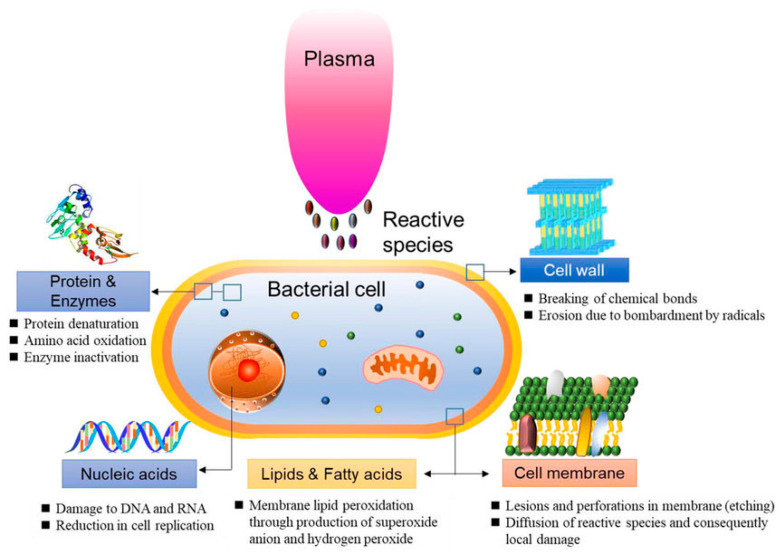
Schematic representation of bacterial reduction induced by CAP.

In 2021, Abbasi et al. [68] examined the effect of CAP on *P. aeruginosa* isolated from burn infections in vitro and in vivo. The research results showed no bacterial growth, as well as wound healing in mice. The study concluded that CAP decreased the expression of the alp gene that is one of the virulence factors of *P. aeruginosa* (Figure 4).

Some studies have described a hybrid treatment for wound healing: CAP therapy combined with antibiotic treatment.

Nguyen et al. [47] applied cold plasma in treating patients with severe COVID-19 who had skin injuries such as burns, pressure ulcers, shingles, and contact or atopic dermatitis. Before the plasma application, all the skin injuries were treated with antibiotics and albumin infusions. After 14 days of cold plasma irradiation, 14/20 patients had complete epithelialization. Previously, when cold plasma irradiation was not applied, these lesions took longer to epithelialize or ulcerated even further, with more exudation despite the use of antibiotics.

In a randomized clinical trial, Stratmann et al. [69] applied CAP therapy on patients with diabetic foot ulcers. All wounds were treated with systemic antibiotics during the study and were Wagner Armstrong Grade 1B or 2B. Eligible wounds were randomized to receive either a placebo or CAP. In this randomized clinical trial, CAP therapy emerged as an efficient treatment in terms of wound surface reduction and wound closure time.

## 4. Discussion

Patients admitted to intensive care units are at high risk of increased morbidity and mortality from skin infections and deep wounds [70]. CAP potentially influences various stages of wound healing by assisting in the activation of microorganisms in the early stage and boosting the proliferation and migration of skin-bound cells afterward.

The studies included in this review of CAP devices employed for biological applications can be divided into two groups, depending on the technology used to generate the plasma. One kind generates plasma within the device, which is then transferred to the treatment area through a carrier gas, such as argon. Techniques include everything from tiny plasma needles to large spotlights.

In the other kind, plasma is created in ambient air using dielectric barrier discharge (DBD) devices at the treatment location by applying high voltage [24].

Three biological effects of CAP are evidenced in the relevant literature: inactivation of a broad spectrum of microorganisms, including multi-drug-resistant microorganisms; increased cell proliferation and increased angiogenesis with a shorter period of CAP treatment; and apoptosis onset with a longer and more intensive treatment period, particularly for tumor cells [23]. What has been observed following treatments with physical plasma is the production of NO, a powerful vasodilator, due to the pH reduction in the treated tissue [71].

Infections can considerably slow the healing of skin wounds, resulting in an increase in healthcare expenses [72]. Likewise, additional expenditures to the care facility for the use of particular devices, suitable dressings, and the commitment of human resources for nursing care are unavoidable [73].

Treatment with CAP is effective in lowering the microbial load [74] and can be applied directly to cells and tissues [75]. Its ability to reduce the bacterial load makes it a valid substitute for antibiotics and a viable therapy to block antibiotic-resistant bacterial strains [75].

The effectiveness of CAP treatment depends on several factors, including device design, treatment time, gas flow and composition, plasma jet intensity and frequency, distance from the sample, and factors such as wound type, extracellular matrix, and exudate [76]. The adaptability of CAP is evident not only in its operating concept but also in its capacity to collaborate with other forms of therapy.

A further advantage of CAP-generating devices is their relatively low production cost [60]. Therefore, accessible, efficient, and less expensive CAP devices can most likely reduce the financial burden imposed on the healthcare budget by conventional treatments. Engineering advances play an important role in the future of CAP wound treatment; the development of portable devices increases access to treatments. Fine tuning of plasma settings can also help optimize delivery to achieve maximum bacterial inhibition and wound healing induction while reducing the frequency of treatment to create an effective and practical solution.

In the future, more possibilities and more benefits of CAP use in clinical practice will be reported, as most studies on CAP have taken place in the last two decades [17,60]. Current analyses of the effects of cold plasma still only focus on cell lines and animal models. Although these experiments are more accessible and easier to conduct, there is a need for further, larger studies specifically with critically ill patients, as to date no studies have been conducted in intensive care settings. Despite differences in research designs, treatment techniques, wound types, and outcome reporting, the data presented by the evaluated studies provide an overview of possible study areas to be implemented, including in the critical care context. CAP has shown great potential for wound healing and may become a promising therapy to replace or supplement existing wound care approaches for critically sick patients. In this regard, the CAP appears to be a very relevant therapeutic alternative when considering the speedier resolution that it may offer for wounds, owing to its potential to reduce the bacterial count in a wound and promote wound healing [77]. Several CAP products have been certified; therefore, further criteria and procedures for plasma treatments should exist.

## 5. Study Limits

The narrative approach used reflects the main limitation of this review. In particular, in order to provide a broad view of CAP use, considering as inclusive a method as possible as is typical of narrative reviews, (a) we did not provide a detailed description of the selection process, which is, therefore, not very replicable, (b) the studies included were not subjected to a qualitative assessment, and (c) the analysis was carried out using a thematic approach established by the researchers in advance [77].

## 6. Conclusions

CAP is effective in many areas of medicine, without significant negative effects on healthy cells. However, its use might have potentially serious side effects and should, thus, be used under expert supervision and with adequate dosages. In this context, this procedure may be standardized and implemented to clinical practice in the future to achieve favorable outcomes in a safe and effective manner. Furthermore, the efficacy of CAP in the treatment of wounds, particularly pressure sores, will allow for a reduction in nosocomial infections, resulting in an improvement in the quality of hospital life and, as a result, a significant reduction in morbidity and mortality associated with HCAIs and bedsores, which also covers the legal implications of this type of therapy. As a result, the focus on the use of CAP and the findings of numerous studies may drive the advancement of technical research, allowing the creation of ever-smaller devices capable of delivering plasma to inside structures.

## Data Availability

The data presented in this study are available on request from the corresponding author.

## References

[1] Zanza C., Romenskaya T., Thangathurai D., Ojetti V., Saviano A., Abenavoli L., Robba C., Cammarota G., Franceschi F., Piccioni A. (2022). Microbiome in Critical Care: An Unconventional and Unknown Ally. Curr. Med. Chem..

[2] Miller P., Smith I.M., White D.M., Taylor D.A., Sherry S.P., Sing R.F. (2016). Wound Management in the ICU. Interventional Critical Care.

[3] Soni K.D., Bansal V., Arora H., Verma S., Wärnberg M.G., Roy N. (2022). The State of Global Trauma and Acute Care Surgery/Surgical Critical Care. Crit. Care Clin..

[4] Klausen M., Heydorn A., Ragas P., Lambertsen L., Aaes-Jørgensen A., Molin S., Tolker-Nielsen T. (2003). Biofilm Formation by Pseudomonas Aeruginosa Wild Type, Flagella and Type IV Pili Mutants: Roles of Bacterial Motility in the Formation of the Flat P. Aeruginosa Biofilm. Mol. Microbiol..

[5] Smolle C., Cambiaso-Daniel J., Forbes A.A., Wurzer P., Hundeshagen G., Branski L.K., Huss F., Kamolz L.-P. (2017). Recent Trends in Burn Epidemiology Worldwide: A Systematic Review. Burns.

[6] Meskini M., Esmaeili D. (2018). The Study of Formulated Zoush Ointment against Wound Infection and Gene Expression of Virulence Factors Pseudomonas Aeruginosa. BMC Complement. Altern. Med..

[7] Bates D.W., Larizgoitia I., Prasopa-Plaizier N., Jha A.K., Research Priority Setting Working Group of the WHO World Alliance for Patient Safety (2009). Global Priorities for Patient Safety Research. BMJ.

[8] Allegranzi B., Bagheri Nejad S., Combescure C., Graafmans W., Attar H., Donaldson L., Pittet D. (2011). Burden of Endemic Health-Care-Associated Infection in Developing Countries: Systematic Review and Meta-Analysis. Lancet.

[9] Burke J.P. (2003). Infection Control—A Problem for Patient Safety. N. Engl. J. Med..

[10] Cassini A., Plachouras D., Eckmanns T., Abu Sin M., Blank H.-P., Ducomble T., Haller S., Harder T., Klingeberg A., Sixtensson M. (2016). Burden of Six Healthcare-Associated Infections on European Population Health: Estimating Incidence-Based Disability-Adjusted Life Years through a Population Prevalence-Based Modelling Study. PLoS Med..

[11] Stegensek Mejía E.M., Jiménez Mendoza A., Romero Gálvez L.E., Aparicio Aguilar A. (2015). Úlceras por presión en diversos servicios de un hospital de segundo nivel de atención. Enferm. Univ..

[12] Edsberg L.E., Langemo D., Baharestani M.M., Posthauer M.E., Goldberg M. (2014). Unavoidable Pressure Injury: State of the Science and Consensus Outcomes. J. Wound Ostomy Cont. Nurs. Off. Publ. Wound Ostomy Cont. Nurses Soc..

[13] Allman R.M., Goode P.S., Patrick M.M., Burst N., Bartolucci A.A. (1995). Pressure Ulcer Risk Factors among Hospitalized Patients with Activity Limitation. JAMA.

[14] Bereded D.T., Salih M.H., Abebe A.E. (2018). Prevalence and Risk Factors of Pressure Ulcer in Hospitalized Adult Patients; a Single Center Study from Ethiopia. BMC Res. Notes.

[15] Graves N., Birrell F., Whitby M. (2005). Effect of Pressure Ulcers on Length of Hospital Stay. Infect. Control Hosp. Epidemiol..

[16] Manzano F., Pérez-Pérez A.M., Martínez-Ruiz S., Garrido-Colmenero C., Roldan D., Jiménez-Quintana M.D.M., Sánchez-Cantalejo E., Colmenero M. (2014). Hospital-Acquired Pressure Ulcers and Risk of Hospital Mortality in Intensive Care Patients on Mechanical Ventilation. J. Eval. Clin. Pract..

[17] Chaves F., Garnacho-Montero J., Del Pozo J.L., Bouza E., Capdevila J.A., de Cueto M., Domínguez M.Á., Esteban J., Fernández-Hidalgo N., Fernández Sampedro M. (2018). Diagnosis and Treatment of Catheter-Related Bloodstream Infection: Clinical Guidelines of the Spanish Society of Infectious Diseases and Clinical Microbiology and (SEIMC) and the Spanish Society of Spanish Society of Intensive and Critical Care Medicine and Coronary Units (SEMICYUC). Med. Intensiv..

[18] Smit J.M., Raadsen R., Blans M.J., Petjak M., Van de Ven P.M., Tuinman P.R. (2018). Bedside Ultrasound to Detect Central Venous Catheter Misplacement and Associated Iatrogenic Complications: A Systematic Review and Meta-Analysis. Crit. Care.

[19] Fischer M., William T., Wohlrab J. (2009). Skin Diseases in Intensive Care Medicine. J. Dtsch. Dermatol. Ges. J. Ger. Soc. Dermatol. JDDG.

[20] Akiki R.K., Anand R.S., Borrelli M., Sarkar I.N., Liu P.Y., Chen E.S. (2021). Predicting Open Wound Mortality in the ICU Using Machine Learning. J. Emerg. Crit. Care Med..

[21] Cox J. (2011). Predictors of Pressure Ulcers in Adult Critical Care Patients. Am. J. Crit. Care Off. Publ. Am. Assoc. Crit. Care Nurses.

[22] Niedźwiedź I., Waśko A., Pawłat J., Polak-Berecka M. (2019). The State of Research on Antimicrobial Activity of Cold Plasma. Pol. J. Microbiol..

[23] VON Woedtke T., Schmidt A., Bekeschus S., Wende K., Weltmann K.-D. (2019). Plasma Medicine: A Field of Applied Redox Biology. In Vivo.

[24] Bernhardt T., Semmler M.L., Schäfer M., Bekeschus S., Emmert S., Boeckmann L. (2019). Plasma Medicine: Applications of Cold Atmospheric Pressure Plasma in Dermatology. Oxidative Med. Cell. Longev..

[25] Dubuc A., Monsarrat P., Virard F., Merbahi N., Sarrette J.-P., Laurencin-Dalicieux S., Cousty S. (2018). Use of Cold-Atmospheric Plasma in Oncology: A Concise Systematic Review. Ther. Adv. Med. Oncol..

[26] Grant M.J., Booth A. (2009). A Typology of Reviews: An Analysis of 14 Review Types and Associated Methodologies. Health Inf. Libr. J..

[27] Baethge C., Goldbeck-Wood S., Mertens S. (2019). SANRA—A Scale for the Quality Assessment of Narrative Review Articles. Res. Integr. Peer Rev..

[28] Martusevich A.K., Surovegina A.V., Bocharin I.V., Nazarov V.V., Minenko I.A., Artamonov M.Y. (2022). Cold Argon Athmospheric Plasma for Biomedicine: Biological Effects, Applications and Possibilities. Antioxidant.

[29] Friedman P.C. (2020). Cold Atmospheric Pressure (Physical) Plasma in Dermatology: Where Are We Today?. Int. J. Dermatol..

[30] Braný D., Dvorská D., Halašová E., Škovierová H. (2020). Cold Atmospheric Plasma: A Powerful Tool for Modern Medicine. Int. J. Mol. Sci..

[31] Nguyen D.B., Lee W.G. (2016). Effects of Ambient Gas on Cold Atmospheric Plasma Discharge in the Decomposition of Trifluoromethane. RSC Adv..

[32] Hoffmann C., Berganza C., Zhang J. (2013). Cold Atmospheric Plasma: Methods of Production and Application in Dentistry and Oncology. Med. Gas Res..

[33] Gurtner G.C., Werner S., Barrandon Y., Longaker M.T. (2008). Wound Repair and Regeneration. Nature.

[34] Velnar T., Bailey T., Smrkolj V. (2009). The Wound Healing Process: An Overview of the Cellular and Molecular Mechanisms. J. Int. Med. Res..

[35] Haertel B., von Woedtke T., Weltmann K.-D., Lindequist U. (2014). Non-Thermal Atmospheric-Pressure Plasma Possible Application in Wound Healing. Biomol. Ther..

[36] Duchesne C., Banzet S., Lataillade J.-J., Rousseau A., Frescaline N. (2019). Cold Atmospheric Plasma Modulates Endothelial Nitric Oxide Synthase Signalling and Enhances Burn Wound Neovascularisation. J. Pathol..

[37] García-Alcantara E., López-Callejas R., Morales-Ramírez P.R., Peña-Eguiluz R., Fajardo-Muñoz R., Mercado-Cabrera A., Barocio S.R., Valencia-Alvarado R., Rodríguez-Méndez B.G., Muñoz-Castro A.E. (2013). Accelerated Mice Skin Acute Wound Healing in Vivo by Combined Treatment of Argon and Helium Plasma Needle. Arch. Med. Res..

[38] Amini M.R., Sheikh Hosseini M., Fatollah S., Mirpour S., Ghoranneviss M., Larijani B., Mohajeri-Tehrani M.R., Khorramizadeh M.R. (2020). Beneficial Effects of Cold Atmospheric Plasma on Inflammatory Phase of Diabetic Foot Ulcers; a Randomized Clinical Trial. J. Diabetes Metab. Disord..

[39] Xu Z., Shen J., Zhang Z., Ma J., Ma R., Zhao Y., Sun Q., Qian S., Zhang H., Ding L. (2015). Inactivation Effects of Non-Thermal Atmospheric-Pressure Helium Plasma Jet on Staphylococcus Aureus Biofilms. Plasma Process. Polym..

[40] Xu G.-M., Shi X.-M., Cai J.-F., Chen S.-L., Li P., Yao C.-W., Chang Z.-S., Zhang G.-J. (2015). Dual Effects of Atmospheric Pressure Plasma Jet on Skin Wound Healing of Mice. Wound Repair Regen. Off. Publ. Wound Heal. Soc. Eur. Tissue Repair Soc..

[41] Gao J., Wang L., Xia C., Yang X., Cao Z., Zheng L., Ko R., Shen C., Yang C., Cheng C. (2019). Cold Atmospheric Plasma Promotes Different Types of Superficial Skin Erosion Wounds Healing. Int. Wound J..

[42] Ernst J., Tanyeli M., Borchardt T., Ojugo M., Helmke A., Viöl W., Schilling A.F., Felmerer G. (2021). Effect on Healing Rates of Wounds Treated with Direct Cold Atmospheric Plasma: A Case Series. J. Wound Care.

[43] Heinlin J., Zimmermann J.L., Zeman F., Bunk W., Isbary G., Landthaler M., Maisch T., Monetti R., Morfill G., Shimizu T. (2013). Randomized Placebo-Controlled Human Pilot Study of Cold Atmospheric Argon Plasma on Skin Graft Donor Sites. Wound Repair Regen. Off. Publ. Wound Heal. Soc. Eur. Tissue Repair Soc..

[44] Frescaline N., Duchesne C., Favier M., Onifarasoaniaina R., Guilbert T., Uzan G., Banzet S., Rousseau A., Lataillade J.-J. (2020). Physical Plasma Therapy Accelerates Wound Re-Epithelialisation and Enhances Extracellular Matrix Formation in Cutaneous Skin Grafts. J. Pathol..

[45] Betancourt-Ángeles M., Peña-Eguiluz R., López-Callejas R., Domínguez-Cadena N.A., Mercado-Cabrera A., Muñoz-Infante J., Rodríguez-Méndez B.G., Valencia-Alvarado R., Moreno-Tapia J.A. (2017). Treatment in the Healing of Burns with a Cold Plasma Source. Int. J. Burns Trauma.

[46] Becker D., Tozo T.C., Batista S.S., Mattos A.L., Silva M.C.B., Rigon S., Laynes R.L., Salomão E.C., Hubner K.D.G., Sorbara S.G.B. (2017). Pressure Ulcers in ICU Patients: Incidence and Clinical and Epidemiological Features: A Multicenter Study in Southern Brazil. Intensive Crit. Care Nurs..

[47] Nguyen T.X., Nguyen D.H., Ho-Man T.P., Bui V.D.A., Phan P.N. (2022). Cold Plasmamed Beam as a Supporting Treatment of Soft Tissue Injuries in Severe COVID-19 Patients: A Preliminary Report. Med. Devices Auckl. N. Z..

[48] Zanza C., Thangathurai J., Audo A., Muir H.A., Candelli M., Pignataro G., Thangathurai D., Cicchinelli S., Racca F., Longhitano Y. (2019). Oxidative stress in critical care and vitamins supplement therapy: “a beneficial care enhancing”. Eur. Rev. Med. Pharmacol. Sci..

[49] Zhao S., Han R., Li Y., Lu C., Chen X., Xiong Z., Mao X. (2019). Investigation of the Mechanism of Enhanced and Directed Differentiation of Neural Stem Cells by an Atmospheric Plasma Jet: A Gene-Level Study. J. Appl. Phys..

[50] Yiu G., He Z. (2006). Glial Inhibition of CNS Axon Regeneration. Nat. Rev. Neurosci..

[51] Katiyar K.S., Lin A., Fridman A., Keating C.E., Cullen D.K., Miller V. (2019). Non-Thermal Plasma Accelerates Astrocyte Regrowth and Neurite Regeneration Following Physical Trauma In Vitro. Appl. Sci..

[52] Longhitano Y., Zanza C., Thangathurai D., Taurone S., Kozel D., Racca F., Audo A., Ravera E., Migneco A., Piccioni A. (2020). Gut Alterations in Septic Patients: A Biochemical Literature Review. Rev. Recent Clin. Trials.

[53] Cotter J.J., Maguire P., Soberon F., Daniels S., O’Gara J.P., Casey E. (2011). Disinfection of Meticillin-Resistant Staphylococcus Aureus and Staphylococcus Epidermidis Biofilms Using a Remote Non-Thermal Gas Plasma. J. Hosp. Infect..

[54] Maisch T., Shimizu T., Li Y.-F., Heinlin J., Karrer S., Morfill G., Zimmermann J.L. (2012). Decolonisation of MRSA, S. Aureus and E. Coli by Cold-Atmospheric Plasma Using a Porcine Skin Model In Vitro. PLoS ONE.

[55] Isbary G., Morfill G., Schmidt H.U., Georgi M., Ramrath K., Heinlin J., Karrer S., Landthaler M., Shimizu T., Steffes B. (2010). A First Prospective Randomized Controlled Trial to Decrease Bacterial Load Using Cold Atmospheric Argon Plasma on Chronic Wounds in Patients. Br. J. Dermatol..

[56] Gallant-Behm C.L., Yin H.Q., Liu S., Heggers J.P., Langford R.E., Olson M.E., Hart D.A., Burrell R.E. (2005). Comparison of in Vitro Disc Diffusion and Time Kill-Kinetic Assays for the Evaluation of Antimicrobial Wound Dressing Efficacy. Wound Repair Regen. Off. Publ. Wound Heal. Soc. Eur. Tissue Repair Soc..

[57] Malone M., Bjarnsholt T., McBain A.J., James G.A., Stoodley P., Leaper D., Tachi M., Schultz G., Swanson T., Wolcott R.D. (2017). The Prevalence of Biofilms in Chronic Wounds: A Systematic Review and Meta-Analysis of Published Data. J. Wound Care.

[58] Isbary G., Heinlin J., Shimizu T., Zimmermann J.L., Morfill G., Schmidt H.-U., Monetti R., Steffes B., Bunk W., Li Y. (2012). Successful and Safe Use of 2° Min Cold Atmospheric Argon Plasma in Chronic Wounds: Results of a Randomized Controlled Trial. Br. J. Dermatol..

[59] Scholtz V., Pazlarova J., Souskova H., Khun J., Julak J. (2015). Nonthermal Plasma--A Tool for Decontamination and Disinfection. Biotechnol. Adv..

[60] Mai-Prochnow A., Bradbury M., Ostrikov K., Murphy A.B. (2015). Pseudomonas Aeruginosa Biofilm Response and Resistance to Cold Atmospheric Pressure Plasma Is Linked to the Redox-Active Molecule Phenazine. PLoS ONE.

[61] Krewing M., Jarzina F., Dirks T., Schubert B., Benedikt J., Lackmann J.-W., Bandow J.E. (2019). Plasma-Sensitive Escherichia Coli Mutants Reveal Plasma Resistance Mechanisms. J. R. Soc. Interface.

[62] Mai-Prochnow A., Clauson M., Hong J., Murphy A.B. (2016). Gram Positive and Gram Negative Bacteria Differ in Their Sensitivity to Cold Plasma. Sci. Rep..

[63] Sader H.S., Farrell D.J., Flamm R.K., Jones R.N. (2014). Antimicrobial Susceptibility of Gram-Negative Organisms Isolated from Patients Hospitalized in Intensive Care Units in United States and European Hospitals (2009–2011). Diagn. Microbiol. Infect. Dis..

[64] Daeschlein G., Scholz S., Ahmed R., von Woedtke T., Haase H., Niggemeier M., Kindel E., Brandenburg R., Weltmann K.-D., Juenger M. (2012). Skin Decontamination by Low-Temperature Atmospheric Pressure Plasma Jet and Dielectric Barrier Discharge Plasma. J. Hosp. Infect..

[65] Joshi D., Ahammad G.V.P.S.Z., Kar S., Sreekrishnan T.R. (2022). Development and Optimization of Low Power Non-Thermal Plasma Jet Operational Parameters for Treating Dyes and Emerging Contaminants. Plasma Sci. Technol..

[66] Das S., Gajula V., Mohapatra S., Singh G., Kar S. (2022). Role of Cold Atmospheric Plasma in Microbial Inactivation and the Factors Affecting Its Efficacy. Health Sci. Rev..

[67] Das S., Gajula V., Mohapatra S., Kar S., Bhatt S., Gautam H., Singh G., Kapil A., Das B., Sood S. Antimicrobial Efficacy of Argon Cold Atmospheric Pressure Plasma Jet on Clinical Isolates of Multidrug-Resistant ESKAPE Bacteria. Proceedings of the IEEE Transactions on Radiation and Plasma Medical Sciences.

[68] Abbasi E., Mehrabadi J.F., Nourani M., Namini Y.N., Mohammadi S., Esmaeili D., Abbasi A. (2021). Evaluation of Cold Atmospheric-Pressure Plasma against Burn Wound Infections and Gene Silencing. Iran. J. Microbiol..

[69] Stratmann B., Costea T.-C., Nolte C., Hiller J., Schmidt J., Reindel J., Masur K., Motz W., Timm J., Kerner W. (2020). Effect of Cold Atmospheric Plasma Therapy vs. Standard Therapy Placebo on Wound Healing in Patients With Diabetic Foot Ulcers: A Randomized Clinical Trial. JAMA Netw. Open.

[70] Gatto V., Scopetti M., La Russa R., Santurro A., Cipolloni L., Viola R.V., Di Sanzo M., Frati P., Fineschi V. (2019). Advanced Loss Eventuality Assessment and Technical Estimates: An Integrated Approach for Management of Healthcare-Associated Infections. Curr. Pharm. Biotechnol..

[71] Schleusser S., Schulz L., Song J., Deichmann H., Griesmann A.-C., Stang F.H., Mailaender P., Kraemer R., Kleemann M., Kisch T. (2022). A Single Application of Cold Atmospheric Plasma (CAP) Improves Blood Flow Parameters in Chronic Wounds. Microcirculation.

[72] Li S., Renick P., Senkowsky J., Nair A., Tang L. (2021). Diagnostics for Wound Infections. Adv. Wound Care.

[73] La Russa R., Ferracuti S. (2022). Clinical Risk Management: As Modern Tool for Prevention and Management of Care and Prevention Occupational Risk. Int. J. Environ. Res. Public Health.

[74] Klämpfl T.G., Isbary G., Shimizu T., Li Y.-F., Zimmermann J.L., Stolz W., Schlegel J., Morfill G.E., Schmidt H.-U. (2012). Cold Atmospheric Air Plasma Sterilization against Spores and Other Microorganisms of Clinical Interest. Appl. Environ. Microbiol..

[75] Izadjoo M., Zack S., Kim H., Skiba J. (2018). Medical applications of cold atmospheric plasma: State of the science. J. Wound Care.

[76] Ehlbeck J., Schnabel U., Polak M., Winter J., von Woedtke T., Brandenburg R., von dem Hagen T., Weltmann K.-D. (2010). Low Temperature Atmospheric Pressure Plasma Sources for Microbial Decontamination. J. Phys. Appl. Phys..

[77] La Russa R., Viola R.V., D’Errico S., Aromatario M., Maiese A., Anibaldi P., Napoli C., Frati P., Fineschi V. (2021). Analysis of Inadequacies in Hospital Care through Medical Liability Litigation. Int. J. Environ. Res. Public Health.

